# Recombinant Env proteins that bind the quaternary-specific, V1/V2-directed PGT antibodies

**DOI:** 10.1186/1742-4690-9-S2-P84

**Published:** 2012-09-13

**Authors:** J Gorman, J McLellan, Y Yang, T Zhou, J Zhu, S Bangaru, N Bayless, P Alff, CP Marshall, PD Kwong

**Affiliations:** 1 National Institutes of Health, Bethesda, MD, USA; 2 Avatar Biotechnologies, Brooklyn, NY, USA

## Background

Antibodies PGT141-145 are broadly neutralizing and recognize a glycan-dependent epitope in the V1/V2 loop, similar to antibodies PG9 and PG16. Collectively, this class of antibodies binds preferentially to the functional viral spike. Although PG9, and to a lesser extent, PG16, bind monomeric gp120s and V1/V2 scaffolds, to date no recombinant env-derived proteins have been identified that bind to antibodies PGT141-145.

## Methods

As a first step toward obtaining structural information of the epitope recognized by PGT141-145, we have created and characterized novel gp140s and epitope scaffolds designed to present the V1/V2 conformation recognized by PGT141-145. To date, over 70 recombinant proteins have been expressed and tested for antibody binding.

## Results

We have identified one V1/V2-scaffold protein that binds to PGT142. The binding is dependent on the HIV-1 strain used in the scaffold. We have also produced trimeric, cleaved gp140 constructs and evaluated them for binding to PGT141-145.

## Conclusion

Proteins that accurately mimic V1/V2 conformations of the functional viral spike are crucial to obtaining structures of the PGT antibodies in complex with their epitopes, and may be ideal immunogens for eliciting broadly neutralizing, V1/V2-directed antibodies in a vaccine setting.

